# The most common errors in automatic ECG interpretation

**DOI:** 10.3389/fphys.2025.1590170

**Published:** 2025-05-22

**Authors:** Krzysztof Kraik, Irena Anna Dykiert, Joanna Niewiadomska, Marta Ziemer-Szymańska, Karolina Mikołajczak, Mikołaj Kreń, Piotr Kukiełka, Adrian Martuszewski, Tomasz Harych, Rafał Poręba, Paweł Gać, Małgorzata Poręba

**Affiliations:** ^1^ Students’ Scientific Association of Cardiovascular Diseases Prevention, Wroclaw Medical University, Wrocław, Poland; ^2^ Students’ Scientific Association of Pathophysiology of the Cardiovascular System “Vide Cor Meum”, Wroclaw Medical University, Wrocław, Poland; ^3^ Institute for Heart Diseases, University Hospital in Wroclaw, Wrocław, Poland; ^4^ Department and Clinic of Rheumatology and Internal Medicine, University Hospital in Wroclaw, Wrocław, Poland; ^5^ VO akut, Centralsjukhuset Kristianstad CSK, Kristianstad, Sweden; ^6^ Department of Pediatric Neurology, Tadeusz Marciniak Lower Silesia Specialist Hospital—Emergency Medicine Center, Wrocław, Poland; ^7^ Department of Environmental Health, Occupational Medicine and Epidemiology, Wroclaw Medical University, Wrocław, Poland; ^8^ Department of Neurology, Specialist Hospital in Walbrzych, Wałbrzych, Poland; ^9^ Department of Tourism and Recreation, Wroclaw University of Health and Sport Sciences, Wrocław, Poland; ^10^ Department of Biological Principles of Physical Activity, Wroclaw University of Health and Sport Sciences, Wrocław, Poland

**Keywords:** ECG, electrocardiography, computer-assisted interpretation, diagnostics, diagnostic errors, arrhythmia

## Abstract

**Introduction:**

The 12-lead electrocardiogram (ECG) is one of modern medicine’s most important and useful diagnostic tests. It is a non-invasive, widely available and relatively cheap method used mainly in the diagnostics of arrhythmias and a variety of other pathologies, including conduction disturbances and ischemia. ECG is also used to evaluate the activity of a pacemaker or other implantable devices. Medical personnel worldwide routinely use the automatic analysis and interpretation of ECGs to support their medical evaluation of patients’ electrocardiograms.

**Methods:**

In the current study, we performed a precise analysis of electrocardiograms from 526 different patients and compared it to the automatic interpretations to determine the frequency of false-positive (overinterpretation) and false-negative (underinterpretation) incorrect interpretations.

**Results:**

It was found that about 39% of ECGs were interpreted incorrectly and amongst the misinterpreted 193 ECG cases, 58% were false-negative, while 57% were false-positive. Additionally, it was revealed that incorrect diagnosis of ischemia (false-positive) was correlated with the body mass index (BMI) of the subjects as well as with the undiagnosed chamber enlargements/hypertrophies. Moreover, we found that in elderly people (>60 years old) a larger number of incorrect diagnoses occurred. The diagnosis of ischemia, which is clinically the most important role of ECG, in our study occurred in 16.1% as a false-positive diagnosis, while in 22.3% ischemia remained unrecognized. Conduction abnormalities were overdiagnosed in 21.8% of cases, while underdiagnosis occurred in 14.5%. Arrhythmias were overdiagnosed in 28% of cases and 17.1% of cases were underdiagnosed.

**Discussion:**

In conclusion, we support the statement that relying on the automatic ECG analysis may lead to misinterpretations, which may mislead the medical staff. Automatic analysis of ECG may contain valuable data, although it requires verification and additional knowledge of electrocardiography in every case to achieve a correct and complete interpretation. Results of our studies should increase the caution among clinicians not to rely fully on the automated analysis. Future perspectives should include the application of AI in algorithms used in ECG analysis by manufacturers and paying more attention to accessing proper feedback from clinicians to device manufacturers.

## 1 Introduction

A hundred years have passed since Willem Einthoven was awarded a Nobel Prize for demonstrating that an electrocardiogram (ECG) could record the electrical activity of the heart, and still the ECG remains one of the most important and useful diagnostic tests in medicine (Kashou et al., 2020). Recent advancements in computing power, wireless technology, digitized data availability, and machine learning have encouraged researchers to invent new methods of ECG evaluation, including artificial intelligence, which is a rapidly developing field of science (Kashou et al., 2020). Currently, many researchers and engineers are trying to improve the diagnostic accuracy of automatic ECG analysis and to develop a perfect tool with fully automated, unbiased and unambiguous ECG analysis (Kashou et al., 2020).

ECG is a non-invasive diagnostic technique often used by physicians worldwide to investigate the severity of numerous cardiovascular diseases, including coronary artery disease and myocardial infarction, as well as numerous arrhythmias. Moreover, it is also used in non-cardiological conditions and preparation for different types of surgical procedures (Surawicz et al., 2009).

Consequently, the ability to interpret ECG is a basic diagnostic skill for the assessment of changes occurring in the cardiac conduction system. Nowadays, devices that record ECG and automatically generate an analysis of the ECG curve, based on the measurements recorded, are an integral part of medical practice. Physicians of various specialties, including cardiologists, often rely on the interpretations provided by this type of device (Milliken et al., 1983; Brailer et al., 1997). Nevertheless, the risk of an incorrect diagnosis should be considered and the physician should reinterpret the ECG (Tsai et al., 2003).

The accuracy of ECG device analysis depends on the type of device and its software. Many algorithms have been designed to perform complex analysis based on digital differential potential recordings (Meyer et al., 2006). Different ECG devices demonstrate differences in the sensitivity of their measurements: the detection of heartbeats and their classification, the analysis of the ECG series, the determination of wave boundaries and the corresponding intervals and further processing, including contour analysis by measuring at specific points in the signal and thus assessing whether any pathologies are present. Therefore, the frequency of misinterpretations concerning single and multiple abnormalities, including arrhythmias and conduction disturbances, is the subject of numerous studies (Guglin and Thatai, 2006).

The study aimed to determine the frequency of misinterpretations made by automated ECG analysis, both false-positive and false-negative diagnoses, to analyze the causes of the mistakes, as well as to identify the factors that cause a particular error to occur more frequently.

## 2 Materials and methods

A total of 526 people were enrolled in the study (216 males and 310 females). The mean age was 57.6 ± 16.4 years, mean weight 78.09 ± 17.32 kg, mean height 167.77 ± 8.84 cm, and mean Body Mass Index (BMI) 27.7 ± 5.2. Adults who reported various cardiovascular complaints were included in the study after giving their informed consent. Table 1 shows the characteristics of the study group.

**TABLE 1:** Characteristics of the studied group.

Parameter	Female	Male	Total patients
Number	*n* = 310	*n* = 216	*n* = 526
Age (years)^a^	57.38 ± 15.67	57.99 ± 17.37	57.63 ± 16.38
Minimum	20	18	18
Maximum	94	98	98
Median	60	61	61
Height (cm)^a^	163.28 ± 6.51	174.26 ± 7.65	167.77 ± 8.84
Minimum	149	156	149
Maximum	181	200	200
Median	163	175	168
Body mass (kg)^a^	72.57 ± 14.83	86.06 ± 17.59	78.09 ± 17.32
Minimum	44	51	44
Maximum	160	180	180
Median	70	85	75
BMI (kg/m^2^)	27.22 ± 5.35	28.27 ± 5.04	27.65 ± 5.25
Minimum	17.30	18.70	17.3
Maximum	56	52	56
Median	26.10	27.50	26.9
BMI: <25 kg/m^2^	37.42% (*n* = 116)	25.00% (*n* = 54)	32.32% (*n* = 170)
BMI: 25–29.99 kg/m^2^	38.06% (*n* = 118)	44.91% (*n* = 97)	40.87% (*n* = 215)
BMI: >30 kg/m^2^	24.52% (*n* = 76)	30.09% (*n* = 65)	26.81% (*n* = 141)
Cardiovascular diseases and comorbidities			
Hypertension	53.87% (*n* = 167)	56.02% (*n* = 121)	54.75% (*n* = 288)
Hypotension	0.65% (*n* = 2)	0.00% (*n* = 0)	0.38% (*n* = 2)
Atrial fibrillation	4.52 (*n* = 14)	7.87% (*n* = 17)	5.89% (*n* = 31)
Coronary disease	8.39% (*n* = 26)	6.02% (*n* = 13)	7.41% (*n* = 39)
Myocardial infarction	7.10% (*n* = 22)	10.65% (*n* = 23)	8.56% (*n* = 45)
Stroke	9.35% (*n* = 29)	6.48% (*n* = 14)	8.17% (*n* = 43)
Atherosclerosis	10.32% (*n* = 32)	6.48% (*n* = 14)	8.75% (*n* = 46)
Diabetes mellitus	12.26% (*n* = 38)	15.74% (*n* = 34)	13.69% (*n* = 72)
Asthma	3.55% (*n* = 11)	3.70% (*n* = 8)	3.61% (*n* = 19)
Thyroid disease	24.52% (*n* = 76)	10.19% (*n* = 22)	18.63% (*n* = 98)
Cancer	8.39% (*n* = 26)	6.02% (*n* = 13)	7.41% (*n* = 39)
Gastrointestinal disease	10.65% (*n* = 33)	8.33% (*n* = 18)	9.70% (*n* = 51)
Respiratory disease	6.13% (n = 19)	3.24% (*n* = 7)	4.94% (*n* = 26)
Urinary tract disease	6.45% (*n* = 20)	10.19% (*n* = 22)	7.98% (*n* = 42)
Osteoporosis	0.32% (*n* = 1)	0.00% (*n* = 0)	0.19% (*n* = 1)

^a^ Arithmetic mean ± standard deviation.

In all participants in the study, a 12-lead resting electrocardiogram with automatic interpretation was performed using the Cardioexpress SL12 device (Spacelabs Healthcare), which is used in clinics and tertiary-level hospitals as well as in scientific centers, and is characterized by clinical accuracy and advanced data analysis. The automatically generated ECG recordings were then interpreted manually, which was used as a basis for categorizing individual interpretations as correct or incorrect.

The ECGs were initially interpreted by medical students associated with the Students’ Scientific Association of Pathophysiology of the Cardiovascular System “Vide Cor Meum.” Students were selected from the group interested in cardiology and electrocardiography and additionally, before entering the project, they were specially trained by cardiologists participating in the study on how to base on the recommendations of the Section of Noninvasive Electrocardiology and Telemedicine of the Polish Cardiac Society (Baranowski et al., 2016a; Baranowski et al., 2016b; Baranowski et al., 2010). In the next step, electrocardiogram interpretations were verified by two cardiologists and the assessments were performed independently and blinded to the automatic interpretation. In case of discrepancies in interpretation, they were resolved by consensus. Incorrect interpretations were divided into overinterpretation or failure to recognize abnormalities in the ECG curve.

In order to establish inter-rater reliability between student and cardiologist interpretation, we performed the *post hoc* analysis of the randomly selected 30 electrocardiograms from the present study and the kappa score was found to be 0,8, which shows a strong level of agreement.

In addition, a self-designed questionnaire was carried out on all study participants. The questionnaire included questions about chronic and past diseases, family history, risk factors for cardiovascular disease (dietary habits, physical activity, smoking, consumption of alcohol and coffee), and use of medication (including analgesics). Written informed consent was obtained from all volunteers taking part in the study. The study was approved by the Local Ethics Committee, No. KB–262/2017.

Statistical analysis of the obtained material was performed using Statistica 13.3 software (StatSoft, Poland). Data distribution was checked with the Lilliefors test. The chi-square test was used to verify the statistical significance of qualitative variables, whereas the non-parametric Mann–Whitney U test was used to assess quantitative variables not characterized by normal distribution. The level of statistical significance was defined at *p* < 0.05.

## 3 Results

The number of correctly interpreted electrocardiograms (ECGs) was 319 (60.5%), while 208 (39.5%) ECG records were incorrectly interpreted, of which 82 in men (38.4% of all men) and 125 in women (40.2% of all women).

In the 193 abnormally interpreted ECGs, we specified the type of abnormal description - failure to recognize pathology in the presence of existing changes in cardiac electrical function (112 records – 58.0% – false-negative result) and recognition of pathology without actual changes in cardiac electrical function (111 records – 57.5% – false-positive result), and then we categorized them into five groups:

• Ischemic features–ST-segment elevation, ST-segment depression (measured at J-point), T-wave changes, occurring in at least two leads;
• Arrhythmia–bradycardia, tachycardia, premature ventricular contraction (PVC), atrial fibrillation (AF), premature supraventricular contraction (PSVC), atrial or ventricular pacing;
• Conduction disturbances–sinoatrial block (SA block), first-degree atrioventricular block (AV I block), second-degree atrioventricular block (AV II block), right bundle branch block (RBBB), left bundle branch block (LBBB), left anterior hemiblock (LAH);
• Hypertrophic heart chambers–ECG signs of atrial or ventricular overload;
• Other–e.g., refraction voltages, cardiac axis.

Table 2 presents types of misinterpreted abnormalities seen in ECGs divided into false-positive and false-negative diagnoses.

**TABLE 2:** Types of misinterpreted abnormalities seen in electrocardiograms divided into false-positive and false-negative diagnoses.

Abnormality	False-positive	False-negative
Ischemic features	16.15% (*n* = 31)	22.3% (*n* = 43)
Conduction disturbances	21.8% (*n* = 42)	14.5% (*n* = 28)
Rhythm disturbances	21.2% (*n* = 41)	17.1% (*n* = 33)
Cavity hypertrophy	2.1% (*n* = 4)	9.3% (*n* = 18)
Other pathologies	2.1% (*n* = 4)	1.6% (*n* = 3)

The incorrect diagnosis of ischemic features correlated with the BMI of the subjects. In this group BMI was significantly higher (mean: 29.76 ± 5.48, median: 30.1) than in case of other misdiagnosed or unrecognized pathologies (mean: 27.04 ± 5.22, median: 26.2). A similar correlation with BMI was found for failure to diagnose ischemic features and failure to diagnose hypertrophic heart chambers such as LVH (left ventricular hypertrophy) and LAE (left atrial enlargement). In the first case there was an association with significantly lower body weight (mean: 26.39 ± 5.11, median: 25.7) than for the other false interpretations (mean: 27.72 ± 5.37, median: 27). In contrast, for unrecognized cardiac chamber hypertrophy there was a correlation, as for falsely recognized ischemic features, with higher BMI (mean: 30.06 ± 5.13, median: 28.2).

Table 3 shows the frequency of incorrect interpretations of automated ECG evaluation regarding the gender category.

**TABLE 3:** Frequency of incorrect interpretations of automated electrocardiogram evaluation regarding the gender category.

Sex	Total	% of total	Correct interpretation	Incorrect interpretation	% of incorrect interpretations	*p*
Female	310	58.9	186	123	39.7	0.7006
Male	216	41.1	133	82	38	

The patient’s gender had no statistically significant effect on the total frequency of errors in automatic ECG assessment (chi-square test with Yates correction *p*-value about 0.74, without Yates correction *p*-value about 0.67). The results of ECG interpretation by the device were statistically significantly impacted by the age of the subjects.

In Table 4 we present the frequency of incorrect interpretations of automated ECG evaluation in groups distinguished based on the criterion of ageing as defined by the World Health Organization (WHO).

**TABLE 4:** Frequency of incorrect interpretations of automated electrocardiogram evaluation in groups distinguished based on the criterion of ageing as defined by the World Health Organization (WHO).

Age subgroup	Total	% of total	Correct interpretation	Incorrect interpretation	% of incorrect interpretations	*p*
<60 years old	249	47.34	170	79	31.73	0.0006
≥60 years old	278	52.85	149	129	46.40	

In groups divided by the median age and concerning the criterion of ageing, which the WHO defines as 60 years of age (<60 years of age, ≥60 years of age), there was a correlation between age and wrong diagnosis (*p* < 0.001, *p* < 0.001, respectively).

Table 5 shows the frequency of incorrect interpretations of automated ECG assessment in groups distinguished by median age.

**TABLE 5:** Frequency of incorrect interpretations of automated electrocardiogram assessment in groups distinguished by median age.

Age subgroup	Total	% of total	Correct interpretation	Incorrect interpretation	% of incorrect interpretations	*p*
<61 years old	258	49.05	174	84	32.56	0.001
≥61 years old	269	51.14	145	124	46.10	

In individual groups, the frequency of abnormalities was as follows: <Me–32.56%, >Me–46.10%, <60 years old–31.73%, ≥60 years old–46.40%.

Table 6 presents the frequency of errors in the automated analysis of ECGs concerning the BMI criterion.

**TABLE 6:** Frequency of errors in the automated analysis of electrocardiograms with regards to the Body Mass Index (BMI) criterion.

BMI subgroup	Total	% of total	Correct interpretation	Incorrect interpretation	% of incorrect interpretations	*p*
Underweight (<18.5 kg/m^2^)	2	0.38	1	1	50.00	0.7737
Normal weight (18.5–24.9 kg/m^2^)	165	31.37	96	69	41.82	
Overweight (25–29.9 kg/m^2^)	215	40.87	130	85	39.53	
Obese (≥30 kg/m^2^)	141	26.81	90	51	36.17	

The subject’s BMI was not significant in the total frequency of errors made by the ECG device during analysis (*p* = 0.47). The percentage of incorrect ECG interpretations in the groups separated by BMI was as follows: underweight subjects (BMI < 18.5) – 50%, normal weight subjects (18.5 ≤ BMI > 25) – 41.82%, overweight subjects (25 ≤ BMI > 30) – 39.53%, obese subjects (BMI ≥ 30) – 36.17%. ANOVA test showed no statistically significant difference in the frequency of device errors between groups (*p* = 0.77).

**FIGURE 1:**
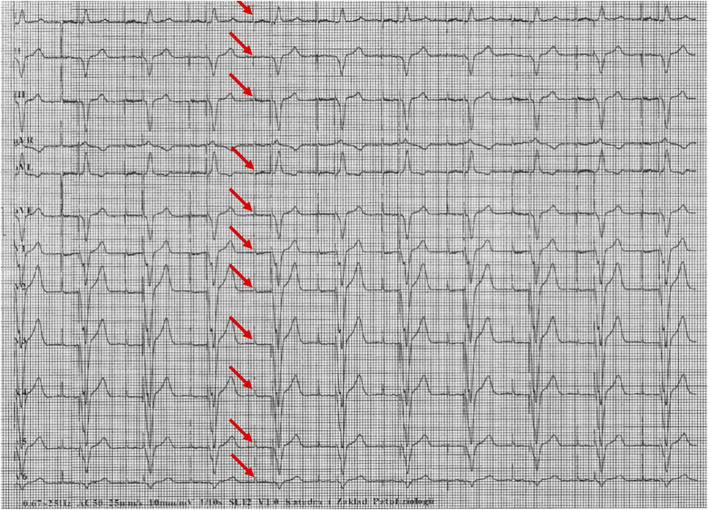
Example of incorrect interpretation by the device: Male, 85 years old. The rhythm was described as atrial flutter with a complete right bundle branch block (25 mm/s, 10 mm/mV). The real diagnosis is dual chamber pacing.

**FIGURE 2:**
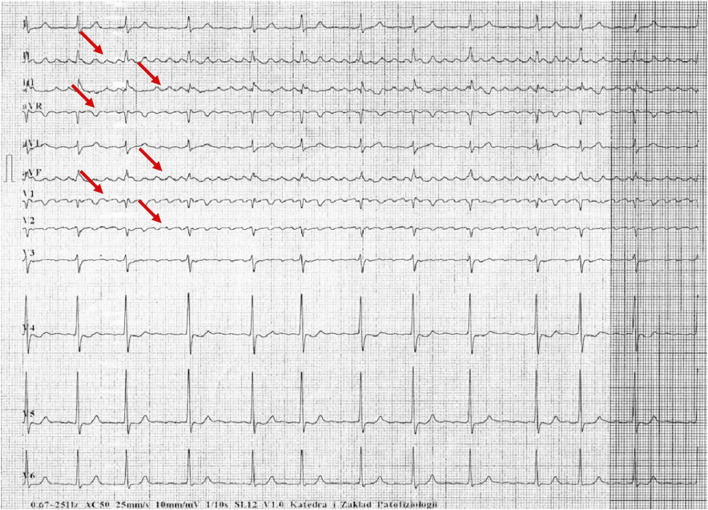
Example of incorrect interpretation by the device: Female, 70 years old. Coarse atrial fibrillation described by the device as sinus rhythm with first-degree atrioventricular block, Mobitz type I second-degree atrioventricular block (Wenckebach) and premature ventricular beats (25 mm/s, 10 mm/mV).

**FIGURE 3:**
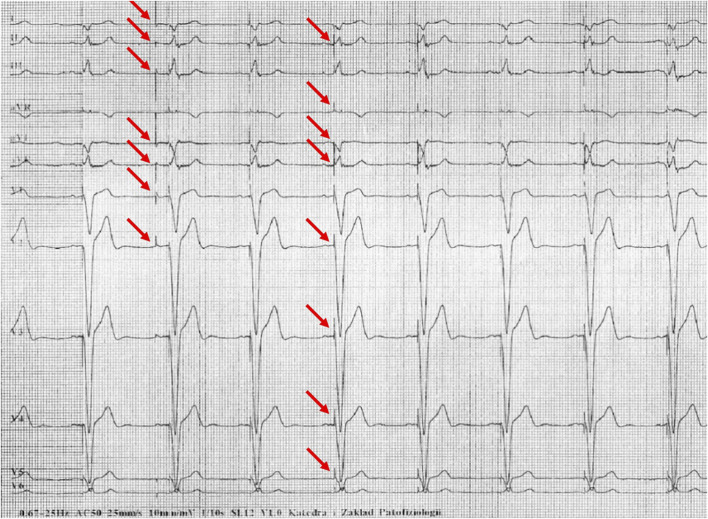
Example of incorrect interpretation by the device: Male, 69 years old. Stimulated rhythm described by the device as complete left bundle branch block and suspected left atrial rhythm (25 mm/s, 10 mm/mV).

**FIGURE 4:**
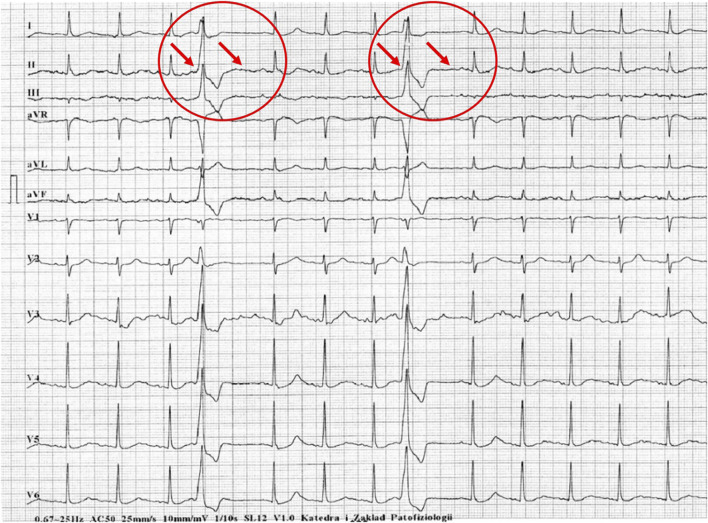
Example of incorrect interpretation by the device: Male, 57 years old. Premature ventricular beats were correctly described by the machine, but the subsequent compensatory pause was interpreted as Mobitz type II second-degree atrioventricular block AV and intraventricular conduction block (25 mm/s, 10 mm/mV).

**FIGURE 5:**
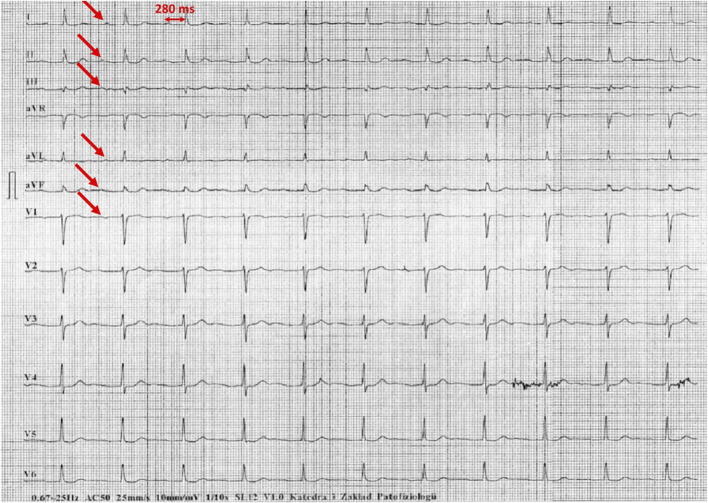
Example of incorrect interpretation by the device: Male, 78 years old. First-degree atrioventricular block described by the device as atrial fibrillation with intraventricular conduction block (25 mm/s, 10 mm/mV).

Figures 1–5 present a sample of ECGs incorrectly interpreted by the device. There are cases of pathologies commonly identified by clinicians. In Figure 1, we identified the dual-chamber pacing with a dual response (DDD) stimulation interpreted as atrial fibrillation and RBBB, so in this case, we have both overinterpretation and failure of recognition. A similar situation is presented in Figure 2, where we found the lack of atrial fibrillation diagnosis and at the same time overinterpretation with type 1 and 2 atrioventricular block. In Figure 3, the situation is presented where, instead of DDD pacing, LBBB and the atrial rhythm were proposed. Moreover, in Figure 4, the compensatory pause after premature ventricular beats was overinterpreted as atrioventricular Mobitz type II block. Finally, in Figure 5, we again identified the lack of proper diagnosis of the first-degree atrioventricular block, together with the overinterpretation of atrial fibrillation.

## 4 Discussion

ECG is one of the most important tests for the initial diagnosis of myocardial infarction by analyzing ST-segment elevation or depression and assessment of T-waves. It is also used to diagnose arrhythmias and sometimes to assess the function of implantable devices such as pacemakers and implantable cardioverter-defibrillators (ICDs). For example, the detection of abnormalities in the ECG is not synonymous with the diagnosis of a recent myocardial infarction, although, in combination with typical symptoms, the patient’s history and comparison with previous ECGs can provide an important indication for further diagnosis and treatment. In addition to the analysis of the ECG, the knowledge and experience of the clinician are crucial.

The analysis of ECG findings made by automated diagnostic devices for the presence of ST-elevation myocardial infarction (STEMI), depending on the type of algorithm used, showed that incorrect diagnoses of STEMI were in a wide range. This range is from 0% to 42% of misinterpretations and from 22% to 42% for not interpreting ECG changes as significant, with the actual presence of features typical for ST-elevation myocardial infarction (Schläpfer and Wellens, 2017). In another publication (Guglin and Thatai, 2006), out of 206 records incorrectly interpreted by the device (out of 2,072), misdiagnoses of myocardial infarction and ischemic features accounted for a total of 7.8% (16 cases). Misdiagnosed ischemia was interpreted in 4 ECGs, all of which were false positives. In addition, the device made errors in the assessment of past myocardial infarctions, not detecting four of them and incorrectly interpreting nine of them. In both cases, this is a significant number of incorrect diagnoses.

In the analysis conducted by the authors of the present study, out of 193 incorrectly assessed ECG recordings, the percentage of incorrectly diagnosed ischemic features was 16.1% (*n* = 31), while for unrecognized ischemic features – 22.3% (*n* = 43). In the data reported, features of past myocardial infarctions were also included. Thus, the results obtained are within relatively wide error limits foreseen for this pathology. Taking into account the fact that device ECG analysis can not only simplify but also interfere with the diagnostic process, we should try to standardize ECG algorithms, improve software and clearly define diagnostic criteria for individual diseases. Cooperation between manufacturers of automated diagnostic devices and physicians is necessary, as well as continuous training of clinicians through active ECG training.

In our study, the total incorrect interpretations made by the device affected about 39% of cases, which gives us a glimpse of the scale on which we can be concerned about the incorrect analysis suggested by the ECG device. In other studies, the majority of abnormal false-positive diagnoses for features of myocardial ischemia occurred in people with higher BMI, and as it is known that obesity can change the appearance of the waves in the ECG, low body weight can also have such an effect (Fraley et al., 2005; Kurisu et al., 2018). However, when the authors divided the participants into groups according to BMI, no correlation was found between errors made by the device and BMI. The mentioned correlation was observed in the division into age groups, where the number of errors in the automatic description was higher in the group of older people. In our study, BMI was also not significantly connected with the total frequency of errors made by the ECG device, even though the number of errors varied between the BMI subgroups.

By suggesting a diagnosis, especially to young, inexperienced physicians, automated diagnostic technologies can result in inappropriate management of an otherwise healthy patient and referral to unnecessary and dangerous treatment.

A significant problem of device-based ECG analysis is also the lack of inclusion of more challenging abnormalities that require assessment by an experienced physician. The analysis performed by ECG devices also does not provide reliable data that would help indicate the site of myocardial ischemia. In most cases, the algorithms make an incorrect assessment of the ST segment and thus the presence or absence of features suggesting infarction or unstable angina due to numerous artifacts in the ECG recording. Another problem is the presence of ST-segment elevation or ST-segment depression for reasons other than myocardial infarction or ischemia, e.g., due to early repolarisation syndrome (ERS) (Schläpfer and Wellens, 2017), cardiac hypertrophy, electrolyte changes, other non-specific causes, etc. (Petrov et al., 2012; Birnbaum and Alam, 2014; Ali et al., 2011).

An important factor impacting further clinical decisions, e.g., in acute coronary syndromes, is time, as prolongation of time may be associated with higher patient mortality, especially in patients belonging to high-risk groups and in those brought to the hospital within 3 h since the first symptoms of ischemia (Larson and Henry, 2008). It is known that time has a significant impact on the outcome of treatment in patients with STEMI myocardial infarction. According to American Heart Association (AHA) guidelines (Collet et al., 2021), it is recommended that it should be as short as possible period (10 min) from the analysis of the initial ECG to the decision to treat. It is important to take into account previous ECG recordings as well as the evolution of ST-segment changes (Larson et al., 2007). Automated ECG analysis may not differentiate ST-segment elevation from early repolarization syndrome, pericarditis or Brugada syndrome. In one of the studies, of 19 ECGs in which the device diagnosed acute myocardial infarction, eight were correctly identified, while the remaining 11 were false positives. Of the false diagnoses, in three cases they were due to early repolarization (Guglin and Thatai, 2006). Even for experienced cardiologists, differentiating a mild form of early repolarization from an acute cardiac event is problematic. This is one of the more common causes of misdiagnosis of fresh myocardial infarction (Larson et al., 2007).

Our study did not include patients with a history of unstable angina, although it is known that sometimes patients may have silent angina–in such cases ST-segment changes analysis may be crucial and contribute to further management. In our study, over 22% of patients with ischemia in ECG were not diagnosed by the algorithm and the ischemia should be defined by ESC guidelines as ST segment depression measured at the J-point by at least 1 mm (−0.5) in two adjacent leads (Thygesen et al., 2019; Wagner et al., 2009). In our study, the device falsely reported ischemic features in 16% of patients–this occurs most frequently in patients with left ventricular hypertrophic changes, but also in younger patients, those training sports and in some cases for unknown reasons (Orlandi et al., 2020; Schocken, 2014; Agrawal et al., 2019).

Jayroe et al. (2009) highlighted in their study the significant impact of external factors on the clinician’s final ECG-based diagnosis. In this study, 116 ECGs containing ST-segment elevation were evaluated by 15 experts. At the same time, false information was provided about the presence of symptoms of acute coronary syndrome in all patients. Study participants were asked to select those records that warranted urgent referral of the patient for percutaneous coronary intervention (PCI). False-positive diagnoses accounted for 12% of the total. It is very possible that the combination of the time factor and the incorrect diagnosis suggested by automated diagnostic systems can be the source of many mistakes in further therapeutic management and the resulting financial and health consequences.

In our study, problems with the correct analysis of heart rhythm were in the third and fourth place in the classification of the frequency of errors in device ECG interpretation. We classified arrhythmia diagnoses by the occurrence of the following descriptions: bradycardia, premature ventricular or supraventricular beats, AF, and additional ventricular and atrial pacing.

Incorrect interpretation of the recordings with the present pacing concerned 5 ECGs and included: misdiagnosis of sinus rhythm with the presence of paced rhythm (DDI (dual-chamber pacing, dual-chamber sensing, inhibited response) mode), misdiagnosis of atrial pacing as atrial flutter, failure to recognize ventricular pacing despite visible pacing peaks, and in two cases misclassification of ventricular pacing as premature ventricular beats. This is one of the most frequent errors according to other authors (Schläpfer and Wellens, 2017; Bae et al., 2012; Smulyan, 2019). In another study (Shah and Rubin, 2007), sinus rhythm was correctly identified in 95% of cases (1,666 correct interpretations out of 1,753 ECGs in which this rhythm was present). According to some authors, AF is the predominant overdiagnosed pathology (Schläpfer and Wellens, 2017; Bae et al., 2012; Bogun et al., 2004). In a study (Bae et al., 2012) including 1,057 ECGs, the device overinterpreted AF in 98 cases and misdiagnosed AF in 119 cases, representing 11.3% and 9.3% of the total number of ECGs analyzed respectively. Particular difficulties occur when bradycardia, tachycardia or artifacts are present because in these cases artifacts may be taken as waves in ECG or sometimes T waves are recognized as QRS complexes making the rhythm rate double. These mistakes are observed in everyday practice as well as in our analysis. Other abnormalities resulting in incorrect AF assessment also include the presence of ventricular pacing and atrial premature beats (Guzman et al., 2020). All of the previously mentioned disorders significantly reduce the chance of an accurate diagnosis of AF, which will mean a delay in the implementation of anticoagulant treatment. Inappropriate therapeutic management may result in an increased risk of stroke if AF is not diagnosed or an increased risk of bleeding associated with inappropriate implementation of anticoagulants when AF is overdiagnosed (Schläpfer and Wellens, 2017; Bae et al., 2012). Also, atrial premature beats are described as the most frequently overdiagnosed arrhythmia. Other frequently overdiagnosed rhythm disorders include premature ventricular beats and atrial flutter (Guglin and Thatai, 2006). In our study, incorrectly diagnosed arrhythmias were, as mentioned before, ranked in third place (41 ECGs – 28%).

In fourth place in our analysis is the failure to diagnose the arrhythmia (17.1% of cases of misdiagnosis by device). According to other researchers, this situation occurs relatively often (Guglin and Thatai, 2006; Shah and Rubin, 2007). Difficulties occur in the case of non-sinus rhythms, bradycardia, tachycardia and artifacts (Bae et al., 2012; Shah and Rubin, 2007). Another possibility is that incorrect pathology is diagnosed–supraventricular or sinus tachycardia instead of AF. In 119 cases out of 1,057 ECGs the device diagnosed sinus tachycardia or supraventricular tachycardia instead of AF (Bae et al., 2012).

Our study revealed a significant frequency of device errors in the assessment of conduction disturbances. Guglin et al.’s study (Guglin and Thatai, 2006) revealed that 86.4% of all conduction disturbances assessment errors were misdiagnosis and misinterpretation of arrhythmias and difficulties in the assessment of patients with a pacemaker. However, in our study, the device incorrectly interpreted conduction disturbances in 36.3% of cases and pacemaker analysis was performed separately. Conduction disturbances included: sinoatrial blocks, atrioventricular blocks, RBBB, LBBB, and LAH. Moreover, our study revealed that misdiagnosed/overdiagnosed conduction disturbances ranked 2nd (42 ECGs – 21.8%), just behind the failure to diagnose ischemia (43 ECGs – 22.3%). According to data presented by Garcia et al. (1981), the sensitivity of conduction disturbance detection by the analyzed algorithm (IBM-Bonner-2) was 93.4%. The most frequently overdiagnosed conduction disturbances are AV I block (6 ECGs), AV II block (6 ECGs), RBBB (4 ECGs), and LBBB (3 ECGs) (Guglin and Thatai, 2006).

In sixth place are unrecognized conduction disturbances (28 ECGs – 14.5%). This is a less common error than the misdiagnosis of conduction disturbances where they are not present. A similar view is expressed by other researchers (Guglin and Thatai, 2006), in whose study the following data can be found: false-positives were found in 19 ECGs (6.5%) and false-negatives in 14 ECGs (4.8%). The group of most frequently unrecognized pathologies included: RBBB (8 ECGs), LBBB (3 ECGs) and AV I block. In a study by other authors (Garcia et al., 1981), the specificity of detecting conduction disturbances was 98.7%, which, in their opinion, was a less frequent error than overdiagnosis of conduction disturbances.

It is important to evaluate the impact of errors in device-based ECG interpretation on further patient treatment. Most authors claim that the physician should confirm the device interpretation because otherwise the patient is exposed to inappropriate treatment and delay in the implementation of effective therapy and the hospital is exposed to additional and unnecessary costs (Bae et al., 2012; Smulyan, 2019; Shah and Rubin, 2007). One of the reasons for the misinterpretations is believed to be the inability of the computer to consider the patient’s clinical condition (Smulyan, 2019). Some authors present the view that physicians should analyze only those ECGs that the device has classified as abnormal. This would make their work much easier and spare time required for ECG interpretation (Hughes et al., 2017). However, this increases the risk of a wrong interpretation. It has been revealed that if a non-cardiology resident has access to a device interpretation that is correct, they are more likely to make an accurate diagnosis, whereas if it is incorrect, the risk of error increases (Tsai et al., 2003). The device interprets ECGs with minor abnormalities more accurately than physicians, but more often makes errors in the analysis of ECGs that are clinically significant (Snyder et al., 2003). It is necessary to continuously educate physicians on ECG interpretation and factors causing the device to misdiagnose (Schläpfer and Wellens, 2017; Bae et al., 2012; Bogun et al., 2004). The use of device-based ECG evaluation is now recommended, although, then the reinterpretation carried out by a physician is required (Smulyan, 2019). It is possible that the more advanced signal analysis techniques could be relevant for future algorithm development.

Future perspectives of this study include two important directions: one for clinicians and the second for manufacturers connected with the creation of software for ECG recorders. Summarizing the results of the present study, when using the automatic ECG analysis, physicians should be conscious of the problem of a lack of full reliance, and in our study, only 60% of electrocardiograms were identified correctly. Out of the incorrectly analyzed ECGs, clinicians may encounter false negative and false positive diagnoses, and in selected cases, even both situations in one ECG, which we presented in the examples in Figures 1–5. We assume that in the next years, new studies should be carried out, including AI-based algorithms, which are now slowly being adopted by manufacturers in new generations of ECG recorders. At the moment physicians using in their practice automated analysis should be aware of the common types of ECG misdiagnoses and they include: 1. Ischemic features misinterpretations (ST elevations/depressions, T wave changes), 2. Arrhythmias misidentifications (bradyarrhythmias, tachyarrhythmias, premature contractions misanalysis), 3. Conduction disturbances (false positive or negative diagnosis), 4. Hypertrophic and overload ECG changes misinterpretations, and 5. Other changes, including pacing analysis problems, mistakes due to high/low voltages and axis misinterpretations. Secondly, the message for manufacturers is also of great importance. Applying more and more advanced novel algorithms makes engineers, IT specialists and physicists less sensitive to the proper feedback from clinicians and simultaneously less open to criticism. It is possible that applying AI may improve the automated analysis in ECG recorders, however, this change follows rather slowly.

The limitation of our study is an analysis of automatic ECG interpretations made by a single device from a particular manufacturer with a single algorithm. Due to this fact, it was impossible to make a comparison of other devices available on the market with separate algorithms in terms of efficiency. Another limitation is that certain comorbidities, including previous myocardial infarction, could have been associated with higher rates of false readings. However, we did not carry out the analysis because the number of patients with selected conditions was relatively low, and it would not be representative. Additionally, the adjustments for multiple comparisons in selected subgroups were not performed, which may increase the probability of type 1 error.

Future studies are required in which similar algorithms used by different manufacturers would be compared on a large patient population.

The data that support the findings of this study are available on request from the corresponding author. The data are not publicly available due to restrictions, e.g., their containing information that could compromise the privacy of research participants.

## Data Availability

The raw data supporting the conclusions of this article will be made available by the authors, without undue reservation.

## References

[B1] Agrawal A. Lu M. Kanjanahattakij N. Jeon H. D. Romero-Corral A. Figueredo V. (2019). ECG clues for false ST-segment elevation myocardial infarction activations. Coron. Artery Dis. 30, 406–412. 10.1097/MCA.0000000000000716 30694822

[B2] Ali S. Goodman S. G. Yan R. T. Budaj A. Fox K. A. A. Gore J. M. (2011). Prognostic significance of electrocardiographic-determined left ventricular hypertrophy and associated ST-segment depression in patients with non–ST-elevation acute coronary syndromes. Am. Heart J. 161, 878–885. 10.1016/j.ahj.2011.02.006 21570517

[B3] Bae M. H. Lee J. H. Yang D. H. Park H. S. Cho Y. Chae S. C. (2012). Erroneous computer electrocardiogram interpretation of atrial fibrillation and its clinical consequences. Clin. Cardiol. 35, 348–353. 10.1002/clc.22000 22644921 PMC6652532

[B4] Baranowski R. Wojciechowski D. Kozłowski D. Kukla P. Kurpesa M. Lelakowski J. (2016a). [Compendium for performing and describing the resting electrocardiogram. Diagnostic criteria describe rhythm, electrical axis of the heart, QRS voltage, automaticity and conduction disorders. Experts' group statement of the Working Group on Noninvasive Ele]. Kardiol. Pol. 74, 493–500. 10.5603/KP.2016.0070 27197010

[B5] Baranowski R. Wojciechowski D. Kozłowski D. Kukla P. Kurpesa M. Lelakowski J. (2016b). [Electrocardiographic criteria for diagnosis of the heart chamber enlargement, necrosis and repolarisation abnormalities including acute coronary syndromes. Experts' group statement of the working group on noninvasive electrocardiology and telemedicine of]. Kardiol. Pol. 74, 812–819. 10.5603/KP.2016.0119 27553353

[B6] Baranowski R. Wojciechowski D. Maciejewska M. (2010). Zalecenia dotyczące stosowania rozpoznań elektrokardiograficznych. Dokument opracowany przez grupę roboczą powołaną przez zarząd sekcji elektrokardiologii nieinwazyjnej i telemedycyny polskiego towarzystwa kardiologicznego. Kardiol. Pol. 68 (Suppl. IV), 335–390. Available at: https://journals.viamedica.pl/polish_heart_journal/article/view/79588/61622.pdf

[B7] Birnbaum Y. Alam M. (2014). LVH and the diagnosis of STEMI - how should we apply the current guidelines? J. Electrocardiol. 47, 655–660. 10.1016/j.jelectrocard.2014.06.001 24997779

[B8] Bogun F. Anh D. Kalahasty G. Wissner E. Bou Serhal C. Bazzi R. (2004). Misdiagnosis of atrial fibrillation and its clinical consequences. Am. J. Med. 117, 636–642. 10.1016/j.amjmed.2004.06.024 15501200

[B9] Brailer D. J. Kroch E. Pauly M. V. (1997). The impact of computer-assisted test interpretation on physician decision making: the case of electrocardiograms. Med. Decis. Mak. 17, 80–86. 10.1177/0272989X9701700109 8994154

[B10] Collet J.-P. Thiele H. Barbato E. Barthélémy O. Bauersachs J. Bhatt D. L. (2021). 2020 ESC guidelines for the management of acute coronary syndromes in patients presenting without persistent ST-segment elevation. Eur. Heart J. 42, 1289–1367. 10.1093/eurheartj/ehaa575 32860058

[B11] Fraley M. A. Birchem J. A. Senkottaiyan N. Alpert M. A. (2005). Obesity and the electrocardiogram. Obes. Rev. 6, 275–281. 10.1111/j.1467-789X.2005.00199.x 16246213

[B12] Garcia R. Breneman G. M. Goldstein S. (1981). Electrocardiogram computer analysis. Practical value of the IBM bonner-2 (V2 MO) program. J. Electrocardiol. 14, 283–288. 10.1016/S0022-0736(81)80010-6 6455484

[B13] Guglin M. E. Thatai D. (2006). Common errors in computer electrocardiogram interpretation. Int. J. Cardiol. 106, 232–237. 10.1016/j.ijcard.2005.02.007 16321696

[B14] Guzman N. R. Escabi J. Babilonia J. R. Martinez-Diaz J. Mirza Z. Cochran M. (2020). Diagnostic accuracy of atrial fibrillation by computerized electrocardiogram analysis versus cardiologist interpretation. J. Am. Coll. Cardiol. 75, 422. 10.1016/S0735-1097(20)31049-4 32000955

[B15] Hughes K. E. Lewis S. M. Katz L. Jones J. (2017). Safety of computer interpretation of normal triage electrocardiograms. Acad. Emerg. Med. 24, 120–124. 10.1111/acem.13067 27519772

[B16] Jayroe J. B. Spodick D. H. Nikus K. Madias J. Fiol M. De Luna A. B. (2009). Differentiating ST elevation myocardial infarction and nonischemic causes of ST elevation by analyzing the presenting electrocardiogram. Am. J. Cardiol. 103, 301–306. 10.1016/j.amjcard.2008.09.082 19166679

[B17] Kashou A. H. May A. M. Noseworthy P. A. (2020). Artificial intelligence-enabled ECG: a modern lens on an old technology. Curr. Cardiol. Rep. 22, 57. 10.1007/s11886-020-01317-x 32562154

[B18] Kurisu S. Nitta K. Sumimoto Y. Ikenaga H. Ishibashi K. Fukuda Y. (2018). Frontal QRS-T angle and World Health Organization classification for body mass index. Int. J. Cardiol. 272, 185–188. 10.1016/j.ijcard.2018.08.060 30172477

[B19] Larson D. M. Henry T. D. (2008). Reperfusion options in ST-elevation myocardial infarction patients with expected delays. Curr. Cardiol. Rep. 10, 415–423. 10.1007/s11886-008-0065-6 18715539

[B20] Larson D. M. Menssen K. M. Sharkey S. W. Duval S. Schwartz R. S. Harris J. (2007). “False-Positive” cardiac catheterization laboratory activation among patients with suspected ST-segment elevation myocardial infarction. JAMA 298, 2754–2760. 10.1001/jama.298.23.2754 18165668

[B21] Meyer C. Gavela J. F. Harris M. (2006). Combining algorithms in automatic detection of QRS complexes in ECG signals. IEEE Trans. Inf. Technol. Biomed. 10, 468–475. 10.1109/TITB.2006.875662 16871713

[B22] Milliken J. A. Pipberger H. Pipberger H. V. Araoye M. A. Ari R. Burggraf G. W. (1983). The impact of an ECG computer analysis program on the cardiologist’s interpretation. A cooperative study. J. Electrocardiol. 16, 141–149. 10.1016/S0022-0736(83)80018-1 6222129

[B23] Orlandi M. Orlandi G. Bini V. Fiorillo C. Becatti M. Stefani L. (2020). The ST segment depression pattern in asymptomatic peri-menopausal Female athletes. Heliyon 6, e04738. 10.1016/j.heliyon.2020.e04738 32923714 PMC7475225

[B24] Petrov D. B. Sardovski S. I. Milanova M. H. (2012). Severe hypokalemia masquerading myocardial ischemia. Cardiol. Res. 3, 236–238. 10.4021/cr222w 28348694 PMC5358138

[B25] Schläpfer J. Wellens H. J. (2017). Computer-interpreted electrocardiograms: benefits and limitations. J. Am. Coll. Cardiol. 70, 1183–1192. 10.1016/j.jacc.2017.07.723 28838369

[B26] Schocken D. D. (2014). Electrocardiographic left ventricular strain pattern: everything old is new again. J. Electrocardiol. 47, 595–598. 10.1016/j.jelectrocard.2014.06.005 25037905

[B27] Shah A. P. Rubin S. A. (2007). Errors in the computerized electrocardiogram interpretation of cardiac rhythm. J. Electrocardiol. 40, 385–390. 10.1016/j.jelectrocard.2007.03.008 17531257

[B28] Smulyan H. (2019). The computerized ECG: friend and foe. Am. J. Med. 132, 153–160. 10.1016/j.amjmed.2018.08.025 30205084

[B29] Snyder C. S. Fenrich A. L. Friedman R. A. Macias C. O’Reilly K. Kertesz N. J. (2003). The emergency department versus the computer: which is the better electrocardiographer? Pediatr. Cardiol. 24, 364–368. 10.1007/s00246-002-0332-z 12457259

[B30] Surawicz B. Childers R. Deal B. J. Gettes L. S. Bailey J. J. Gorgels A. (2009). AHA/ACCF/HRS recommendations for the standardization and interpretation of the electrocardiogram: part III: intraventricular conduction disturbances: a scientific statement from the American heart association electrocardiography and arrhythmias committee, council on clinical cardiology; the American College of cardiology foundation; and the heart rhythm society. Endorsed by the international society for computerized electrocardiology. J. Am. Coll. Cardiol. 53, 976–981. 10.1016/j.jacc.2008.12.013 19281930

[B31] Thygesen K. Alpert J. S. Jaffe A. S. Chaitman B. R. Bax J. J. Morrow D. A. (2019). Fourth universal definition of myocardial infarction (2018). Eur. Heart J. 40, 237–269. 10.1093/eurheartj/ehy462 30165617

[B32] Tsai T. L. Fridsma D. B. Gatti G. (2003). Computer decision support as a source of interpretation error: the case of electrocardiograms. J. Am. Med. Inf. Assoc. 10, 478–483. 10.1197/jamia.M1279 PMC212785 12807810

[B33] Wagner G. S. Macfarlane P. Wellens H. Josephson M. Gorgels A. Mirvis D. M. (2009). AHA/ACCF/HRS recommendations for the standardization and interpretation of the electrocardiogram: part VI: acute ischemia/infarction: a scientific statement from the American heart association electrocardiography and arrhythmias committee, council on clinical cardiology; the American College of cardiology foundation; and the heart rhythm society. Endorsed by the international society for computerized electrocardiology. J. Am. Coll. Cardiol. 53, 1003–1011. 10.1016/j.jacc.2008.12.016 19281933

